# Multidisciplinary Management of Morbidities Associated with Chronic Graft-Versus-Host Disease

**DOI:** 10.46989/001c.124926

**Published:** 2024-10-21

**Authors:** Rahul Shah, Danielle Murphy, Melissa Logue, James Jerkins, Andrew Jallouk, Kassim Adetola, Olalekan Oluwole, Reena Jayani, Eden Biltibo, Tae K. Kim, Salyka Sengsayadeth, Wichai Chinratanalab, Carrie Kitko, Bipin Savani, Bhagirathbhai Dholaria

**Affiliations:** 1 Division of Cancer Medicine The University of Texas MD Anderson Cancer Center; 2 Department of Medicine Vanderbilt University Medical Center; 3 Division of Hematology/Oncology, Department of Medicine Vanderbilt University Medical Center; 4 Division of Hematology/Oncology, Department of Pediatrics Vanderbilt University Medical Center

**Keywords:** chronic GVHD, allogeneic stem cell transplant

## Abstract

Chronic graft-versus-host disease (cGVHD) represents a common long-term complication after allogeneic hematopoietic stem cell transplantation (HSCT). It imposes a significant morbidity burden and is the leading cause of non-relapse mortality among long-term HSCT survivors. cGVHD can manifest in nearly any organ, severely affecting the quality of life of a transplant survivor. While the mainstay of treatment has remained systemic immunosuppression with glucocorticoids, progress has been made within the last few years with approvals of three oral agents to treat steroid-refractory cGVHD: ibrutinib, ruxolitinib, and belumosudil. Iatrogenesis contributes a significant portion of the morbidity experienced by patients with cGVHD, primarily from glucocorticoids. This review highlights the myriad impacts of cGVHD, including and beyond the traditional organ systems captured by the National Institutes of Health Consensus Criteria, including iatrogenic complications of long-term immunosuppression. It presents the implications of cGVHD and its treatment on cardiovascular and metabolic health, bone density, endocrine function, sexual health, and ocular and pulmonary disease and outlines a framework around the comprehensive multidisciplinary approach for its evaluation and management.

## Introduction

### Incidence and Risk Factors

Chronic graft-versus-host disease (cGVHD) is a common long-term complication following allogeneic hematopoietic stem cell transplantation (HSCT), occurring in 30 to 70% of patients.^1–3^ The condition and its treatment impose a significant non-relapse morbidity and mortality burden among survivors of allogeneic HSCT.^1^ While acute GVHD affects epithelial tissues, typically the skin, liver, and gastrointestinal tract, classically presenting with rash, cholestatic liver injury, and diarrhea, respectively, cGVHD can cause autoimmune-like manifestations in virtually any organ system and is often multisystem at presentation.^3,4^

Several independent donor, recipient, and transplant-related factors have been associated with the development of cGVHD. Older patient age at transplant, prior acute GVHD, HLA (human leukocyte antigen) disparity, use of a mobilized peripheral blood stem cell graft, and a female donor graft for a male recipient are all associated with increased risk of cGVHD.^3,5,6^

### Pathophysiology

There have been significant advances in understanding the cGVHD pathophysiology over the last decade, though it remains much less understood than acute GVHD. Organ fibrosis and dysfunction because of alloimmune tissue injury underpin the clinical manifestations of cGVHD.^3^ The pathogenesis of cGVHD has been conceptualized as occurring in three phases.^3^ The first phase is characterized by tissue damage mediated by the transplant conditioning regimen, acute GVHD, and infectious processes, which mobilize antigen-presenting cells of the innate immune system and activate T-cells.^3^ The adaptive immune response and chronic inflammation represent the second phase, propagating alloreactive T- and B-cells.^3^ The final phase involves fibroblast proliferation, dysregulated tissue repair and fibrosis, and organ dysfunction.^3^

### Impact on Survivorship

HSCT has become safer over time, with a decrease in non-relapse mortality rates over the last few decades due to improvements in transplantation practices.^7,8^ At the same time, there has been an increase in the incidence of cGVHD, which still is a significant contributor to late mortality in patients after allogeneic HSCT (8-10). In a large retrospective study of 7489 patients who were leukemia-free at one year after allogeneic transplantation, Boyiadzis et al. reported that cGVHD significantly increases the risk of transplant-related mortality, conferring a 2.4 times greater risk compared to those without cGVHD.^9^ Furthermore, cGVHD was also associated with an increased overall mortality risk, with a relative risk of 1.56.^9^ In a prospective longitudinal analysis of 937 patients with cGVHD and a median follow-up of four years, DeFillip et al. found that cGVHD itself was the most common cause of non-relapse mortality at 38%, most commonly because of organ failure or infection.^10^

In addition to its impact on survival, cGVHD can dramatically affect the physical and emotional quality of life during cancer survivorship by reducing global and organ-specific levels of function.^11^ A survey analysis of 1377 patients by Lee et al. found that, compared to patients without cGVHD and those with resolved cGVHD, patients with moderate or severe cGVHD had a worse quality of life and physical symptom burden, in addition to reporting higher rates of prescription medications for pain, anxiety, and depression.^12^ Moreover, immunosuppressive agents, particularly glucocorticoids, used to treat cGVHD can impose significant toxicities that worsen the quality of life in these patients. Clinicians often underestimate the detrimental impact to quality of life that cGVHD can have on these patients, highlighting the need for a thoughtful multidisciplinary approach that can diagnose, grade, and treat the heterogeneous manifestations of cGVHD and the toxicities from its treatment.^11^ With the undeniable impact on morbidity and mortality, patients with cGVHD are best served when cared for by a multidisciplinary team of transplant physicians, nurses, advanced practice providers, primary care physicians, subspeciality physicians with experience with HSCT survivors, and allied health professionals.

## Evaluation and Treatment of Chronic GVHD

### Diagnosis and Staging

The National Institutes of Health (NIH) Consensus Conference introduced standardized criteria in 2005, later revised in 2014, to diagnose and grade the organ-specific and global severity of cGVHD.^2,13^ The diagnosis is made when there is at least one clinical sign of cGVHD or at least one distinctive manifestation in any of the eight target organs made through biopsy, laboratory, or imaging without other non-GVHD causes.^2^ A scoring system measures severity using a scale from zero to three in the skin, mouth, eyes, gastrointestinal tract, liver, lungs, joints/fascia, and genitourinary tract using clinical signs and symptoms, laboratory values, organ-specific testing (e.g., pulmonary function testing) and specialist evaluation (e.g., ophthalmologic exam).^2^ A global severity score of mild, moderate, or severe is also calculated, considering the number of organs involved and the severity score.^2^

While the NIH consensus criteria offer a convenient and standardized approach to the diagnosis and grading of cGVHD, it does not fully capture the impact of cGVHD on long-term cardiovascular outcomes, for example, or the toxicity from cGVHD treatment such as osteoporosis or adrenal insufficiency. This review explores the lesser-discussed impacts of cGVHD and its treatment on the longevity and well-being of transplant patients. **Table 1** summarizes the manifestations, evaluation, and management of the cardiovascular, bone, endocrine, sexual health, ocular, and pulmonary impacts of cGVHD and its treatment discussed in this review.

**Table 1.** Manifestations, Evaluation, and Management of Multimorbidity Associated With Chronic Graft-Versus-Host Disease and its Treatment.

**System**	**Manifestations**	**Evaluation/Monitoring**	**Management**
**Cardiovascular**	Insulin resistance, post-transplant diabetes mellitus Dyslipidemia Accelerated atherosclerosis	Lipid profile Fasting blood sugar and A1c measurement	Cardio-oncology referral Endocrinology referral Consideration of lipid-lowering therapies Nutrition and exercise therapy
**Bone**	Osteopenia and osteoporosis Fragility fracture Osteonecrosis of the jaw (ONJ) Avascular necrosis (AVN) of bone	Vitamin D level Dual-energy x-ray absorptiometry imaging Magnetic resonance imaging if concerned for ONJ and AVN of bone	Weight bearing and resistance exercise Calcium and vitamin D supplementation Bisphosphonate therapy Endocrinology referral for advanced osteoporosis Orthopedics referral for AVN of bone for arthroplasty
**Endocrine**	Adrenal insufficiency Symptomatic hypothyroidism Premature gonadal failure Women: Premature menopause, hypoestrogenism, vulvovaginal atrophy Men: Symptoms of low testosterone, erectile dysfunction	Thyroid function testing Measurement of hormones in hypothalamic-pituitary-adrenal axis	Endocrinology referral Hormone replacement therapy Stress dose steroids when acutely ill
**Sexual Health**	Women: vulvovaginal lesions, vulvar pain, vaginal dryness, dyspareunia, post-coital bleeding, decreased fertility Men: balanoposthitis, lichen sclerosis-like changes, phimosis, urethral meatal stenosis, Peyronie’s disease, erectile dysfunction, decreased fertility	Gynecological exam and potential biopsy to rule out superimposed or secondary cause in women Urological exam	Reproductive endocrinology referral Gynecology referral Urology referral Topical steroids and calcineurin inhibitors Topical estrogen for women
**Ocular Health**	Keratoconjunctivitis sicca: dry eyes, burning, photosensitivity, foreign body sensation Conjunctivitis, conjunctival fibrosis Corneal ulceration or perforation Glaucoma Cataracts	Ophthalmologic evaluation: slit lamp exam, corneal fluorescein staining, Schirmer’s test, tonometry	Ophthalmology referral Artificial tears Topical steroids and calcineurin inhibitors Scleral lenses Phacoemulsification for cataracts Glaucoma therapy
**Pulmonary**	Obstructive lung disease: bronchiolitis obliterans syndrome Cough, wheezing, dyspnea Hypoxemia and respiratory failure Opportunistic pulmonary infections, bronchiectasis	Pulmonary function testing High resolution chest computed tomography Bronchoscopy	Pulmonary rehabilitation Fluticasone, azithromycin, montelukast Inhaled bronchodilators Prophylactic antimicrobials

### Treatment

The treatment paradigm for cGVHD revolves around therapy to mitigate the symptoms and morbidity associated with immune-mediated tissue damage, while awaiting immune tolerance after transplantation that allows for gradual withdrawal of immunosuppressive therapy.^14^ In patients with milder manifestations of cGVHD, local therapies can be used for both symptom control and disease modification.^14^ Steroid or calcineurin inhibitor eye drops, and artificial tears are commonly used for eye involvement^15^; topical steroids can be used for skin involvement^16^; and oral steroid rinses are often used for oral involvement with the addition of sialagogues if xerostomia is present.^17^ In patients with genital cGVHD, in addition to topical steroids and calcineurin inhibitors, the use of topical estrogen therapy can improve vaginal dryness, atrophy, dysuria, and dyspareunia.^18^

High dose systemic glucocorticoids are the mainstay of therapy for moderate-to-severe cGVHD. Prednisone is typically started at 0.5 to 1 mg/kg daily, used either alone or in combination with calcineurin inhibitors, e.g., tacrolimus or cyclosporine.^14^ Symptoms and manifestations of cGVHD should be closely followed and graded at each clinical encounter to assess therapeutic response to first-line therapy and to inform the timing of the gradual taper of glucocorticoids.^14^ Prior randomized trials comparing other agents (e.g., ibrutinib, mycophenolate mofetil, and azathioprine) in combination with prednisone in the front-line setting have not demonstrated superiority over prednisone alone.^19–21^ There remains a strong need for the development of steroid-minimizing and steroid-sparing therapies for the initial treatment of cGVHD.

Half of patients experience steroid-refractory (SR) cGVHD and require treatment with second-line therapy.^22^ There are three FDA-approved options for SR cGVHD: ibrutinib, ruxolitinib, and belumosudil.^23–25^ Approved in 2021, ruxolitinib is a Janus-associated kinase 1 and 2 inhibitor which is often favored as the initial therapy for SR cGVHD over ibrutinib, a Bruton tyrosine kinase inhibitor and the first FDA-approved second-line therapy for cGVHD in 2017.^23,25,26^ More recently, belumosudil, a rho-associated coiled-coil-containing protein kinase-2 (ROCK2) inhibitor, was approved as third-line treatment of SR cGVHD.^24^ In addition to FDA-pharmacologic agents, extracorporeal photopheresis (ECP) is also used as therapy beyond first-line in cGVHD, particularly when skin, mouth, lung, and liver are involved.^16,27,28^

As a common post-transplant complication which often requires long-term management, cGVHD can add a significant financial burden on health care systems in low- and middle-income countries which perform allogeneic HSCT.^29,30^ While glucocorticoids generally remain widely accessible and first-line for cGVHD worldwide, equitable access and availability of ruxolitinib, ibrutinib, and belomosudil for SR cGVHD may be constrained in many countries.^29,30^ More accessible immunosuppressive drugs that are commonly used second-line after steroids include calcineurin inhibitors (tacrolimus and cyclosporine), methotrexate, mycophenolate mofetil, mTOR inhibitors (sirolimus and everolimus), and rituximab.^31^ The first approved tyrosine kinase inhibitor imatinib, currently off patent, has also been used to good effect and should be considered in patients with SR cGVHD.^31,32^

### Morbidity from immunosuppression

Iatrogenesis contributes a significant portion of the morbidity experienced by patients with cGVHD.^1,14^ Thus, a key goal in the treatment of patients with cGVHD involves close monitoring for iatrogenic effects of immunosuppressive therapies, particularly of glucocorticoids. Infection due to both cGVHD-associated immune dysregulation and long-term use of immunosuppression is the leading cause of non-relapse mortality in patients with cGVHD,^10^ thus necessitating the use of multi-agent anti-microbial prophylaxis in this patient population. Moreover, cGVHD is associated with delayed immune reconstitution after transplant, which also increases infection risk.^33^ Regimens for antimicrobial prophylaxis vary depending on transplant center.^34^ In general, patients with cGVHD on high dose steroids warrant prophylactic agents against fungal organisms with azole antifungals, against *Varicella* and *Herpes simplex* viruses with acyclovir or valacyclovir, and against *Pneumocystis* with trimethoprim-sulfamethoxazole, dapsone, atovaquone, or pentamidine.^1,35^ Prophylactic therapy that includes coverage of pneumococcus should also be considered. Routine surveillance labs for CMV reactivation should also be performed.^1,35^ While the benefit is still uncertain, replacement therapy with intravenous immunoglobulin is often administered in the setting of hypogammaglobulinemia, often at a serum IgG<400 mg/dL.^36^

## Metabolic abnormalities and cardiovascular health

HSCT survivors are at elevated risk for cardiovascular disease.^37–40^ A recent retrospective study of 6677 HSCT survivors found that this group had sixty percent greater odds of developing coronary heart disease compared to sibling controls.^40^ Cardiovascular disease represents a growing share of the morbidity and mortality among transplant survivors, particularly as these patients are living longer and as the average age of transplant is rising over time. cGVHD contributes to the risk of developing many of the factors associated with cardiovascular disease.

### Metabolic syndrome and cardiovascular risk factors

Metabolic syndrome comprises a collection of risk factors including insulin resistance, dyslipidemia, hypertension and central obesity, which are associated with an increased risk of diabetes and cardiovascular disease which, in turn, are associated with a risk of cardiovascular and all-cause mortality.^41–43^ Survivors of allogeneic HSCT, particularly those with cGVHD, are at elevated risk for the development of the components of metabolic syndrome.^38,39,41,44–47^

The development of post-transplant diabetes mellitus (PTDM) after HSCT is common.^45–48^ While its pathogenesis remains incompletely understood, it is thought to be at least partially a result of the immunosuppressive medications used to treat both acute and chronic GVHD, such as calcineurin inhibitors and glucocorticoids.^45,47^

Dyslipidemia is often observed after allogeneic HSCT, particularly in those with cGVHD.^37–39^ Insulin resistance and diabetes, radiation, immunosuppressive medications and cGVHD of the liver all play a key role in its development.^38,39^ Cyclosporine and tacrolimus are both associated with increases in serum lipid levels.^38^ Prolonged glucocorticoids can contribute to insulin resistance and may also lead to increase in lipid levels. Moreover, thyroid disorders, particularly hypothyroidism, occur at higher rates in transplant survivors with cGVHD and have been associated with dyslipidemia and elevated risks of cardiovascular events.^49–51^

It is well established that systemic inflammatory syndromes can accelerate atherosclerosis and the development of coronary heart disease.^52^ There is evidence that cGVHD as a chronic inflammatory disorder itself can involve endothelium and cause vascular inflammation and injury, which can promote atherogenesis.^37^

### Management of Metabolic and Cardiovascular Complications

Promoting the longevity of survivors of allogeneic HSCT necessitates a comprehensive approach for the prevention, detection, and management of cardiovascular risk factors and metabolic syndrome. Cardio-oncology has recently emerged as a subspecialty of cardiology that is focused on preventing and managing the cardiovascular complications of cancer and its therapy. As patients with cGVHD are more likely to be diagnosed with the components of metabolic syndrome compared to those without cGVHD, early involvement of cardio-oncologists during the transplant evaluation process and during survivorship is critical. Cardiovascular risk factors need to be aggressively managed, as transplant survivors with cGVHD are at elevated risk for major adverse cardiovascular events, including early mortality. Lipid lowering therapy, particularly statin medications and novel agents such as PSCK-9 inhibitors, should be considered to manage those with elevated lipid profiles.^38^ Drug-drug interventions, particularly with statins and immunosuppressive therapy, are important considerations, which would benefit from the collaboration between transplant physicians and cardio-oncologists.^38^

In the age of novel non-insulin anti-diabetic agents, management of hyperglycemia and PTDM may benefit from involvement from an endocrinologist. Glucagon-like peptide-1 (GLP-1) receptor agonists and sodium-glucose cotransporter-2 (SGLT-2) inhibitors are both agents that have been associated with reductions in cardiovascular mortality and they may eventually play a role in the management of cardiovascular disease and diabetes after HSCT.^53^ Further study is needed to establish guidelines for the evidence-based treatment with these novel drugs in the post-transplant population.

### Lifestyle, Nutrition, and Exercise Therapy

The involvement of allied health professionals such as registered dietitians and physical and occupational therapists is critical in the optimization of cardiovascular risk in patients with cGVHD. As patients with cGVHD experience metabolic syndrome, malnutrition, and sarcopenia at high rates, registered dietitian aid in identifying macro- and micro-nutrient deficiencies and optimizing diet and caloric intake in the setting of dysgeusia and oral GVHD.^54–56^ Exercise interventions and involvement from physical and occupational therapists are important in promoting exercise capacity, physical conditioning, and quality of life in patients with cGHVD.^57,58^ The IRENE-G study is an ongoing randomized clinical trial which is the first that evaluates the impact of a combined resistance exercise and nutritional intervention in patients with acute or chronic GVHD on quality of life, GVHD symptom burden, and performance status.^59^

Patients with cGVHD are at higher risk for developing subsequent cancers due to prolonged immunosuppressant therapy.^60,61^ Thus, behavioral and lifestyle modifications are paramount not only to promote cardiometabolic health and protect organ function, but to reduce the risk of subsequent malignancy during long-term survivorship. In addition to being a strong risk factor for cardiovascular disease, smoking is also associated with pulmonary disease and development of numerous malignancies.^62,63^ Patients should avoid passive and active smoking exposure as well as all tobacco products. Moreover, the importance of a balanced, nutritious diet should not be overlooked, as it can be protective against ischemic heart disease after HSCT.^64^ Patients should be thoroughly counseled on a diet rich in fruits, vegetables, and whole grains and the minimization of processed foods, red meat, and sugar.^64,65^ With a dose-dependent risk of alcohol intake on cancer risk, patients should be also counseled on the avoidance of alcohol.^65,66^

## Bone Health

Survivors of allogenic HSCT with cGVHD are at increased risk for the development of bone loss and osteoporosis,^67^ placing them at higher risk for fragility fracture.^68^ There are several factors that influence this predisposition, including pre-transplant and post-transplant comorbidities. Modifying bone density allows for an avenue through which to improve the survivorship care of patients after allogeneic transplantation.

### Risk factors for osteoporosis

Risk factors for osteoporosis pre-transplant include female gender, post-menopausal state, lower body weight, diabetes mellitus, malnutrition, sedentary lifestyle, smoking, excessive alcohol intake, vitamin D deficiency, limited sun exposure, hypogonadism, pre-existing bone mineral abnormalities, older age, and medications including glucocorticoids and chemotherapy.^67^ Patients with severe cGVHD may be at an increased risk for osteoporosis due to co-existing risk factors including immune dysregulation, premature menopause, weight loss, secondary hypogonadism, reduced mobility and lack of physical activity, and prolonged use of immunosuppressive therapies.^67,68^ Ovarian insufficiency is common following allogeneic HSCT, which places women at a particularly increased risk.^69,70^

Medications commonly used in this patient population that are associated with increased risk of osteoporosis include immunosuppressive agents such as glucocorticoids, calcineurin inhibitors, and chemotherapy, specifically myeloablative conditioning.^67,70^ Reduced intensity regimens have generally been associated with less organ toxicity; however, the impact on bone health remains not well understood.^68^ Cumulative dose and duration of therapy have been found to be strongly associated with osteoporosis.^67,71^ Glucocorticoids are well known for their adverse effects related to bone loss. High doses are often used in the treatment of cGVHD, and both the total cumulative dose and duration of glucocorticoid therapy contribute to the risk of developing decreased bone mineral density.^70,71^ The use of steroid-sparing agents such as ruxolitinib and belumosudil, among other treatments, have the potential to limit steroid use and slow bone loss.^70^

### Prevention, monitoring, and treatment

The most rapid bone loss occurs in the first 6-12 months after transplantation.^70,72^ Those with pre-transplant risk factors are at an even higher risk. Physical activity, supplemental calcium and vitamin D, and consideration of estrogen replacement therapy for affected women are known preventive measures in the post-transplant period.^72^ Recommended supplemental calcium is 1000 mg/day for age ≤50 years and 1200 mg/day for those ≥51 years, and recommended supplemental vitamin D is 800-1000 IU/day.^73^

Dual-energy x-ray absorptiometry (DEXA) imaging is recommended to screen and assess bone loss. Survivors should be monitored with a DEXA scan within one year of HSCT; however, earlier screening should be considered for those identified as at substantial risk.^73,74^ There is some evidence to suggest that DEXA measurement prior to HSCT may be beneficial in identifying those at substantial risk prior to chemotherapy and immunosuppressive therapy exposure.^68,72^

Treatment for osteopenia and osteoporosis are similar to prevention strategies. Patients should be educated on the role of physical exercise including weight-bearing, resistance, and muscle strengthening exercises, and lifestyle changes such as limitation of excessive tobacco and alcohol intake to reduce and prevent bone loss.^68^ Bisphosphonate therapy should be initiated for those with diagnosed osteoporosis; however, some studies suggest prophylactic bisphosphonate use may be beneficial.^68,72,74^ Secondary causes of osteoporosis should be identified and treated, including vitamin D deficiency, hypogonadism, and thyroid disorders.^68,70,72^ The involvement of an endocrinologist should be considered when treating advanced osteoporosis and other endocrinopathies which may contribute to decreased mineral bone density.

### Toxicities from treatment

Intravenous bisphosphonate therapy with zoledronic acid is generally well tolerated. Oral bisphosphonate therapies are well known for their gastrointestinal disturbances that often limit compliance.^75^ Bisphosphonate therapies should be used with caution in patients with known renal impairment and are contraindicated in patients with an estimated glomerular filtration rate<35.^68^ Patients should be screened for renal disfunction prior to bisphosphonate administration.

Osteonecrosis of the jaw (ONJ) is one of the most widely reported adverse effects of bisphosphonate therapies.^75^ The risk is associated with prolonged, high dose, and intravenous treatment. Risk factors for the development of ONJ include invasive oral procedures, dental disease, diabetes, concurrent use of medications such as glucocorticoids, and presence of infection.^75,76^ Patients should be screened prior to administration for recent dental work and postponement of therapy should be considered for those with recent extractions or periodontal surgeries or implants.^75^

Avascular necrosis (AVN) of bone is also a debilitating morbidity after allogeneic HSCT which is caused by impaired blood flow to bone and subsequent necrosis and collapse.^77^ Steroid therapy is a significant dose-dependent risk factor associated with the development of bone AVN.^77–79^ In one prospective study of 207 patients after autologous and allogeneic HSCT with a median follow-up period of twenty-six months, twelve patients developed AVN and all but one also had cGVHD.^68^ The hip is the most affected joint in a vast majority of causes, but the knee, wrist, and ankle may also be involved.^78^ Clinicians should possess a high index of suspicion in any new joint pain in a patient with cGVHD, particularly those on longstanding steroids. Pain is the first sign of AVN of bone, which can sometimes by detected on plain film.^78^ However, magnetic resonance imaging is the diagnostic modality of choice.^78^ Early orthopedic involvement is key for management to reduce long-term loss of physical function in these patients, with total joint arthroplasty being the definitive management.

## Endocrine Effects

Chronic GVHD and its treatment can cause many endocrinopathies like adrenal insufficiency, thyroid dysfunction, growth impairment, and gonadal failure.

### Adrenal

Suppression of the hypothalamic-pituitary-adrenal axis is well-recognized in patients with cGVHD, namely as a result of the high dose steroids necessitated in its treatment.^50^ Prolonged use of steroids in the treatment of cGVHD suppresses ACTH production by the pituitary gland and can culminate in secondary adrenal insufficiency. As patients are often prescribed steroids for many months, caution must be exercised in their taper. Sudden cessation or quick de-escalation of steroids can induce an acute adrenal crisis, particularly as these patients are at substantial risk for severe infections and hospitalization, and may necessitate stress dose steroids for those who have been on chronic steroids.^50^

### Thyroid

Thyroid dysfunction is commonly observed in patients after allogeneic transplantation, particularly in those with cGVHD.^49,50^ Savani et al. found that nearly 40% of long-term survivors of allogeneic HSCT develop either overt or subclinical hypothyroidism, occurring more commonly in patients receiving prolonged immunosuppressive therapy for cGVHD.^49^ Therefore, thyroid function testing should be regularly obtained alongside other laboratory testing in long-term transplant survivorship clinics, particularly as the symptoms of hypothyroidism may not be readily distinguishable from other pathology. In addition to symptoms of hypothyroidism such as fatigue, weight gain, cold intolerance, and depressed mood, hypothyroidism also contributes to metabolic and cardiovascular disease, making early identification and treatment of hypothyroidism important in the management of a transplant survivor. Thyroid function testing should be performed at regular intervals for those after allogeneic transplantation.

### Premature gonadal failure

cGVHD also holds important implications for the fertility of long-term transplant survivors, particularly as a considerable proportion of adult patients undergoing allogeneic transplantation are in their second or third decade of life.^8,80^ In addition to the toxic effects of conditioning regimens, cGVHD contributes to gonadal dysfunction which impairs fertility in both male and female patients.^50^

In women, cGVHD is associated with premature ovarian insufficiency, which holds important implications for fertility, sexual function, cardiometabolic disease, and bone health.^50,69^ Women after allogeneic transplantation can develop hypergonadotropic hypogonadism, with elevated levels of serum LH and FSH levels, depressed levels of 17β-estradiol, Δ4-androstenedione, testosterone,and DHEAS, with the degree of ovarian insufficiency more severe in women with cGVHD.^69^ Consequently, ovarian and uterine volumes are lower in women with cGVHD compared to those without.^69^ Premature ovarian insufficiency can present with symptoms of early menopause, such as vasomotor symptoms and genitourinary symptoms of dyspareunia and dysuria due to vaginal dryness and atrophy in combination with direct vulvovaginal effects of cGVHD.^81^ Early menopause is also a well-documented risk factor for cardiovascular events.^82^

Hypogonadism is also common in men after allogeneic transplantation, which can lead to sexual dysfunction and infertility. Low testosterone production that can be seen in patients with chronic GVHD has been postulated to be a result of chronic immunosuppressive therapy imposing a inhibitory effect on the pituitary-gonadal axis.^50,83^ Low levels of testosterone can present with changes in body hair and testes size, erectile dysfunction, fatigue, loss of libido, and reductions in muscle mass, as well as reductions in bone mineral density.^83^ cGVHD has also been associated with azoospermia.^84^

### Management of endocrine disorders

cGVHD and its management with steroids can lead to long-term endocrinopathies, necessitating early referral to endocrinology as soon as the diagnosis is suspected, as the dosing of hormone replacement requires careful longitudinal monitoring. In addition to managing adrenal insufficiency, thyroid dysfunction and gonadal failure, the treatment of steroid-induced diabetes would also benefit from close involvement from an endocrinologist in the era of novel anti-diabetic drugs, many of which can improve patients’ long-term cardiovascular outcomes. Moreover, the nuanced discussion of the relative risks and benefits of hormone replacement therapy warrants involvement of an endocrinologist.

## Sexual Health and Fertility

Genital cGVHD has a significant impact on the sexual health and function of women and men. Sexual dysfunction is reported by most patients after allogeneic transplantation and is strongly associated with the presence of genital cGVHD.^85,86^

Sexual health remains underreported by patients and underappreciated by clinicians,^87^ putting the onus on the patient’s multidisciplinary team to proactively address sexual and reproductive health to best promote the quality of life of transplant survivors. Promoting sexual health with cGVHD warrants the multidisciplinary involvement of gynecology, urology, reproductive endocrinologists, and other fertility specialists.

### Women

cGVHD of the genital tract occurs more frequently in women than men,^85^ and is characterized by vulvovaginal involvement (e.g., erosions, ulceration, adhesions, stenosis, fibrosis, lichen sclerosis) that can present as vulvar pain, vaginal dryness, dyspareunia, and post-coital bleeding, which can impair sexual function.^88,89^ Gynecologists play a significant role in the management of chronic vulvovaginal GVHD not only for early detection through gynecologic examination but for exclusion of other pathology, such as genital infections or cancers.^89,90^ Topical steroids, cyclosporine and estrogen are mainstays of treatment and can improve symptoms. Vaginal dilators may be required to manage stenosis.^90^ As previously mentioned, endocrine dysfunction, particularly hypogonadism, can also derange sexual function, libido, and fertility. In patients with hypoestrogenism and symptoms of premature menopause, systemic estrogen replacement may be beneficial for symptom control.^90^ Options for hormone replacement include oral or transdermal 17β-estradiol or conjugated equine estrogen.^91^ Importantly, concomitant progesterone is mandatory in women who still have a uterus to prevent endometrial hyperplasia in the setting of unopposed estrogen.^91^ Progesterone formulations include oral micronized progesterone or oral medroxyprogesterone acetate.^91^ While estrogen replacement can improve genitourinary systems and quality of life, caution should be exercised in certain patients. System estrogen replacement is typically contraindicated in patients with a history of coronary heart disease, thrombosis, thrombophilia, stroke, breast and endometrial cancer.^81,92^ Women of reproductive age who are anticipated to undergo allogeneic transplant should be referred to a reproductive specialist to discuss options for fertility preservation.

### Men

Genital cGVHD in men presents as genital skin changes, most commonly as balanoposthitis (inflammation of the glans penis), lichen sclerosis-like changes and phimosis, as reported in a retrospective cohort of 155 male transplant survivors.^93^ Other manifestations of genital cGVHD in men are urethral meatal stenosis and Peyronie’s disease, a fibrotic disorder of the penis which leads to abnormal curvature and pain during erections, both of which warrants referral to a urologist for consideration of procedural management.^90,93,94^ Erectile dysfunction is also reported by most men after allogeneic transplant, and more commonly in men with cGVHD,^93^ likely from multifactorial reasons such as cardiovascular disease, hypogonadism and anatomical dysfunction.^95^ In terms of fertility preservation, post-pubertal men should be referred to a reproductive specialist before transplantation if possible, as patients can experience impaired spermatogenesis even before transplant, which may be further exacerbated if they develop cGVHD.^83^

## Eye health: ocular cGVHD

Ocular manifestations of cGVHD are prevalent and can involve nearly any of the ocular structures, including periorbital skin, conjunctiva, cornea, lens, lacrimal structures, sclera, uvea and retina.^15,96^ Ocular cGVHD occurs in 40-60% of patients and 60-90% of those with cGVHD have ocular symptoms, thereby contributing a significant symptom burden in patients after allogeneic HSCT.^15^ Ocular symptoms are frequently the first manifestation of cGVHD, most often presenting with keratoconjunctivitis sicca or dry eye syndrome due to immune-mediated inflammation and fibrosis of the lacrimal glands.^15^ Patients can present with symptoms of dryness, foreign body sensation, burning, and photosensitivity.^15^ Conjunctival involvement with conjunctivitis and fibrosis also occurs in around 10% of patients with cGVHD.^15^ Corneal involvement ulceration or perforation is also possible, either because of the direct inflammatory involvement of the cornea or inadequate protective lubrication from keratoconjunctivitis sicca.^15,96,97^

The prolonged use of steroids to manage both acute and chronic GVHD following allogenic transplantation also contributes to an increased risk of iatrogenic complications such as the increased risk of developing glaucoma and cataracts.^98–100^

### Ophthalmologic evaluation

The ability to maintain sight is a critical aspect of preserving a high quality of life during transplant survivorship. Thus, in addition to routine eye examinations, any particular concern of ocular involvement of cGVHD should prompt early referral to an ophthalmologist with experience in ocular GVHD for a thorough evaluation with slit lamp exam and other specialized clinical tests as indicated, such as with corneal fluorescein staining, Schirmer’s test, tonometry, among others.^96^ Early detection and treatment of the ocular manifestations of cGVHD is critical to minimize the risk of permanent vision loss. Ophthalmologists versed in GVHD also supervise the use of topical therapies, such as with lubricants and anti-inflammatory drops (e.g., corticosteroid, tacrolimus, cyclosporine), scleral lenses, and procedural interventions which are the mainstay of ocular cGVHD management, as systemic therapy rarely impacts ocular symptoms.^15,96^

While ocular hypertension during systemic steroid therapy can frequently resolve after cessation of therapy, some patients will need close monitoring of intraocular pressures and even dedicated glaucoma therapy.^100^ Moreover, a considerable proportion of patients with cGVHD will require phacoemulsification for cataracts,^99^ making the longitudinal role of the ophthalmologist paramount in the management of patients after allogeneic transplant.

## Pulmonary disease

Pulmonary involvement of cGVHD is common, and classically manifests as bronchiolitis obliterans syndrome (BOS), which is characterized by immune-mediated destruction of the small airways and fixed airway obstruction which in-turn culminates in an obstructive lung disease. The presentation of BOS is variable, ranging from asymptomatic disease to cough, wheezing, and dyspnea.^101^ Its progression can culminate in chronic respiratory failure, oxygen dependence, and end-stage lung disease.^101^ BOS occurs in up to 14% of patients with cGVHD and portends a significant increase in late mortality in patients after allogenic transplantation.^101,102^ While five-year survival over the last decade has improved due to better therapies and supportive care, it remains low at around 50%,^101^ which raises the importance of early detection of BOS, which can improve long-term pulmonary outcomes.^103^ Routine screening pulmonary function remains critical in the early detection of pulmonary cGVHD, with some centers recommending screening PFTs every three months in the first two years after transplant.^101^ The diagnosis of BOS requires a FEV1/FVC ratio of <0.7 and a depressed FEV1 percent predicted of <75% with ≥10% decline over two years, with the exclusion of other causes.^2^ High resolution computed tomography imaging of the chest can be helpful in detecting evidence of air trapping, small airway thickening, and bronchiectasis, which can be seen in BOS, as well as in excluding other pulmonary pathology.^2^

There are a few unique considerations in the management of BOS. In addition to systemic immunosuppression, the combination of inhaled fluticasone, azithromycin and montelukast (FAM) is now commonly used in the treatment of BOS due to evidence that it improves symptom burden and quality of life, while leading to reductions in systemic steroid exposure.^101,104^ While reduction in pulmonary function is irreversible in BOS, supportive therapy with bronchodilators has also been shown to improve symptoms and pulmonary rehabilitation has been shown to improve exercise tolerance.^101^ As superimposed pulmonary infections are particularly common in patients with BOS, antimicrobial prophylaxis is critical in those with BOS if on long-term immunosuppression, with coverage of *Pneumocystis jirovecii*, *Streptococcus*, and fungal organisms.^101^ Involvement from pulmonologists is key, particularly as bronchoscopy may be necessary, as atypical infections are common in patients with BOS.^101^ Vaccinations should also be pursued when possible post-transplant, especially against pneumococcus. However, some caution must be taken with regard to the likelihood of a protective response in patients on chronic immunosuppression and with known immune dysfunction. In the case of end-stage lung disease with worsening hypoxemia, patients should be referred for lung transplant evaluation,^105^ though early time post-transplant (<2 years) may limit patient eligibility.

## Care coordination and health information technology

**Figure 1.**
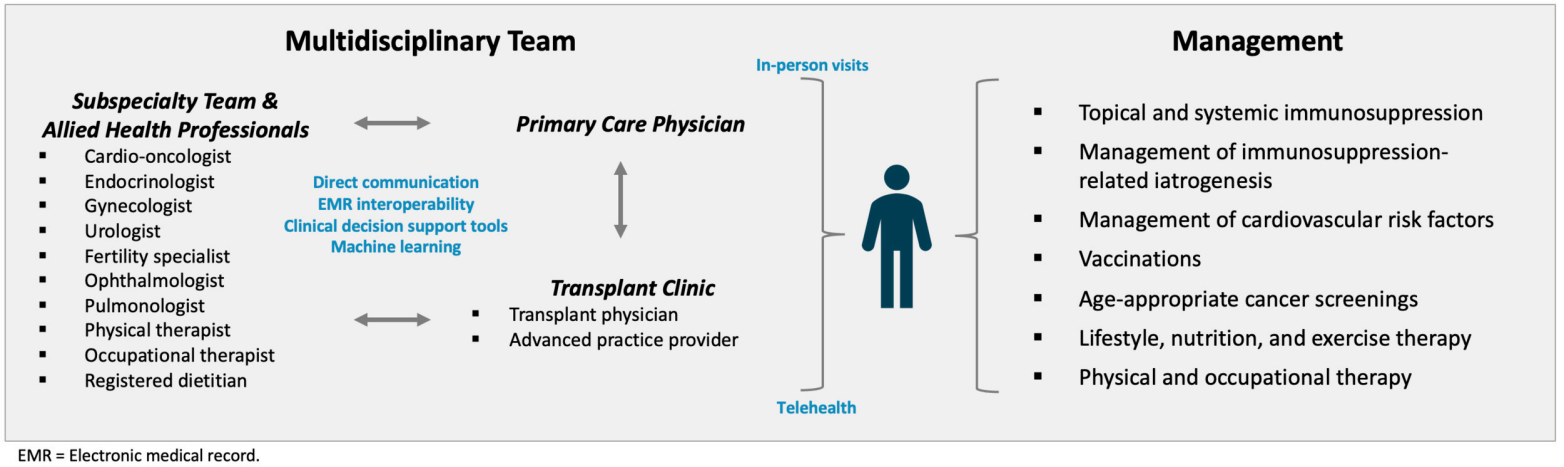
Model for the Multidisciplinary Management of the Patient with Chronic Graft-versus-Host Disease

Seamless care coordination across the many clinicians involved in the care of a long-term transplant survivor is critical for the effective management of cGVHD. A large proportion of transplant survivors live a significant distance from their transplant centers, making the local primary care physician a vital access point for initial triage of symptoms, labs, screening diagnostic tests (e.g., age-appropriate cancer screenings, DEXA), vaccinations, and face-to-face exams for the patient with cGVHD.^106–108^ Thus, transplant physicians should work to develop a direct line of communication between the transplant center and the primary care physician to facilitate the shared care of their mutual patient. Between regular in-person follow-up visits for specialized diagnostics, grading of cGVHD, and titration of immunosuppression, transplant centers must also embrace telehealth as a modality to maintain convenient, patient-centered, and reliable long-term follow-up in the months and years after transplantation. Young adult patients and those who live far from transplant centers may particularly benefit from telehealth, who may find it difficult to attend regular long-term care visits.^106^ Early involvement of subspecialty clinicians is also a key requisite in the successful management of patients in a long-term transplant clinic, particularly as grading of cGVHD, such as with the ophthalmologic and gynecologic exams, or management of treatment iatrogenesis (e.g., diabetes and osteoporosis) often warrants subspecialist expertise. Improvements in the diagnosis and management of cGVHD cannot occur in the siloes of transplant centers; EMR interoperability represents another key pillar of the high quality management of cGVHD in order to enable better the two-way communication from the primary care physician to the transplant physician and subspecialist and back.^109^ Leveraging health information technology with electronic medical record (EMR) decisional support tools and machine learning for the prognostication and diagnosis of cGVHD represents a novel paradigm that is currently being explored in stem cell transplant clinics and warrant greater investment and study.^109–111^ With the increasing popularity of artificial intelligence in healthcare, these technological advances may provide another avenue through which to make allogenic HSCT safer and more effective. **Figure 1** presents the comprehensive care model for the multidisciplinary approach to the management of the transplant survivor with cGVHD.

## Conclusion

cGVHD and toxicities from its treatment dramatically impact the longevity and wellbeing of survivors of allogeneic HSCT. As cGVHD can impact virtually every organ system, transplant survivors are best served in a long-term survivorship clinic that integrates a comprehensive and multidisciplinary approach for the evaluation and management of cGVHD, with a focus on minimizing the iatrogenic complications of immunosuppression. While progress in the treatment of cGVHD has been made in the last few decades, further advancement is required to continue to make allogenic transplantation safer, more accessible, and better tolerated.

### Disclosure of Competing Interests

Andrew Jallouk: Consulting for Kite/Gilead.

Tae K. Kim: Consulting for Agenus and Immunobiome.

Carrie Kitko: Advisory board for Incyte Therapeutics.

Bipin Savani: Editor-in-Chief of *Clinical Hematology International.*

Bhagirathbhai Dholaria: Institutional research funding from Janssen, Angiocrine, Pfizer, Poseida, MEI, Orcabio, Wugen, Allovir Adicet, BMS, Molecular Templates; Consultancy/Advisor for MJH BioScience, Arivan Research, BEAM therapeutics, Janssen, ADC therapeutics, Roche.

Rahul Shah, Danielle Murphy, Melissa Logue, Kassim Adetola, Olalekan Oluwole, Reena Jayani, Eden Biltibo, Salyka Sengsayadeth, Wichai Chinratanalab report no pertinent competing interests.

### Ethics Approval

This review article was exempt from ethics approval as no patient data was collected or utilized.

### Author Contribution Statements

Conceptualization: Rahul Shah, Bhagirathbhai Dholaria

Methodology: Rahul Shah, Bhagirathbhai Dholaria

Writing, original draft preparation: Rahul Shah, Danielle Murphy

Writing, review and editing: Rahul Shah, Danielle Murphy, Melissa Logue, James Jerkins, Andrew Jallouk, Kassim Adetola, Olalekan Oluwole, Reena Jayani, Eden Biltibo, Tae K. Kim, Salyka Sengsayadeth, Wichai Chinratanalab, Carrie L. Kitko, Bipin N. Savani, Bhagirathbhai Dholaria

Supervision: Bhagirathbhai Dholaria
